# Involving people with lived experiences in the study of a behavioral stress-recovery e-intervention for myocardial infarction patients younger than 55 with cardiac distress: a study protocol

**DOI:** 10.1186/s40900-025-00795-z

**Published:** 2025-10-27

**Authors:** Niclas Almén, Ulf O. E. Elofsson, Claudia T. Lissåker, Claes Held, Henrik Nygård, Erik M. G. Olsson

**Affiliations:** 1 Department of Women’s and Children’s Health, Uppsala University, Uppsala, Sweden; 2 Department of Psychology and Social Work, Mid Sweden University, Östersund, Sweden; 3 Department of Physical Activity and Health, The Swedish School of Sport and Health Sciences, Stockholm, Sweden; 4 Karolinska Institutet, Institute of Environmental Medicine, Stockholm, Sweden; 5 Department of Medical Sciences, Cardiology, Uppsala University, Uppsala, Sweden; 6 Uppsala Clinical Research Center, Uppsala University, Uppsala, Sweden

**Keywords:** Myocardial infarction, Younger adults after myocardial infarction with cardiac distress (CD), Patient research partner (PRP), Co-design, Stress recovery, Cardiac distress (CD), Psychological intervention, Patient and public involvement and engagement (PPIE), eHealth, Internet intervention

## Abstract

**Background:**

Public and patient involvement and engagement (PPIE) is increasingly valued for improving the quality and relevance of health research. Patient research partners (PRPs) offer lived experience of a previous myocardial infarction and cardiac distress that can enhance study design and implementation. This protocol describes one out of four studies in a larger project aiming to adapt and evaluate a stress recovery intervention for adults (aged < 55) after myocardial infarction with cardiac distress.

**Objective:**

The primary aim of this study is to explore PRPs’ perceived impact on the design, implementation, and evaluation of the internet-delivered behavioral stress recovery intervention Balance in Everyday Life (iBEL), and to describe the process of their involvement in research activities. This study employs a PPIE approach to systematically involve PRP: s throughout all phases of the research project.

**Methods:**

Five to eight PRPs with lived experiences of a previous myocardial infarction and cardiac distress will participate in a series of structured online workshops across all phases of the project. They provide feedback on intervention materials and study procedures. Data are collected using impact logs, semi-structured interviews, and questionnaires, and will be analyzed using thematic analysis.

**Discussion:**

This study is expected to generate insights into how sustained PRP involvement can be integrated into behavioral intervention research. It will highlight how PRPs influence decision-making processes in real time, and how this aligns with scientific and practical considerations.

**Conclusion:**

The present study provides insights into the perceived impact of PRPs on the design, implementation, and evaluation of the iBEL intervention, highlighting both effective aspects and challenges of patient involvement, and offering guidance for integrating PRP input in behavioral intervention research.

**Clinical trial registration:**

Not applicable. This study does not involve a clinical trial requiring registration.

**Supplementary Information:**

The online version contains supplementary material available at 10.1186/s40900-025-00795-z.

## Abstract (plain language summary)

Younger people who have had a heart attack (also known as a myocardial infarction) often experience emotional problems such as anxiety, fear of another heart event, or difficulties returning to work. These problems are sometimes referred to as cardiac distress. Although cardiac medical treatments (such as surgery and medication) have improved, many people who have had a heart attack struggle with such distress long after the event. This paper describes a part of a larger research project that aims to adapt and evaluate a stress recovery program that could help individuals who have experienced a heart attack before the age of 55 years and subsequently experience cardiac distress. A program that will be delivered online to make it more accessible. In the study described in this paper we focus on involving people from the target group, individuals under 55 who have had a heart attack, in the research process. These individuals are called *Patient Research Partners* (PRPs). They will join structured online workshops with researchers throughout the entire project. These workshops are dedicated to the research project and are distinct from traditional steering group activities, which are separate. Their task is to give feedback on the program, study materials, and how the studies are carried out. We want to understand how PRPs influence the project and what value their involvement brings. To do this, we will collect feedback in several ways, including interviews, surveys, and workshop notes. The findings will help us learn how to involve patients in ways that are more aligned with established principles of meaningful patient and public involvement and engagement, including early involvement (e.g., participation in protocol development, intervention design, and input regarding outcome measures), influence on study decisions, and ongoing collaboration throughout the research process, and how such involvement can improve the quality and practical relevance of healthcare programs.

**Supplementary Information:**

The online version contains supplementary material available at 10.1186/s40900-025-00795-z.

## Introduction

This paper presents the study protocol for one of four studies in a larger research project, focusing specifically on the methodology of involving patients as research partners in the research process. Patient and public involvement and engagement (PPIE), which entails the active participation of individuals from the public or specific target groups in various stages of research, has gained increasing recognition for its value in health-related studies. A closely related approach that has also informed the design of the present study is co-production and co-design, where individuals from the target group collaborate fully and equally with researchers in shaping the research [1]. While our study draws inspiration from co-production principles, it does not adopt them in their entirety. We emphasize active involvement and meaningful collaboration with our Patient Research Partners (PRPs) [2], but without fully implementing the equal partnership model that characterizes co-production. PRP contribute unique perspectives, lived experiences, and insights that can enhance the relevance, impact, and practical application of research findings [3]. Unlike traditional research subjects who are passively studied, PRPs actively collaborate with the research leaders from study design to data analysis and dissemination, providing patient perspectives and ensuring that the research aligns more closely with the needs and realities of people with lived experiences [4].

### Myocardial infarction and the need for psychological interventions

Cardiovascular disease (CVD) remains the leading cause of death globally and the primary cause of disability-adjusted life years lost [5]. Despite advancements in medical treatments and decreasing overall myocardial infarction (MI) rates, the proportion of younger patients (< 55 years) with MI has increased [6]. Psychological/emotional distress, often referred to as cardiac distress (CD), is common among MI survivors and includes anxiety, depression, and disease-specific concerns such as fear of reinfarction, lifestyle adjustments, and social isolation [7]. Younger MI patients frequently experience heightened distress due to the impact on family and work life, which may further increase their risk of reinfarction and compromise their quality of life [8]. CD is also a known risk factor for recurrent CVD events, making effective psychological interventions essential [5, 9].

Returning to work is important for working-age individuals who have experienced an MI, as it reduces financial insecurity, increases self-esteem, and minimizes long-term sick leave and social isolation [10]. However, many working-age individuals who have experienced an MI struggle with the demands of professional and family life due to their reduced physical and psychological capacities, and emotional distress often complicates their return to work [11]. Psychological interventions that address this distress can play a vital role in rehabilitation by supporting emotional well-being, resilience, and recovery processes.

Although emotional distress can affect people of all ages after an MI, younger patients appear to be particularly vulnerable. In a recent national registry study including more than 50,000 individuals, younger patients (< 55 years) had significantly higher odds of reporting emotional distress both two months and one year after a first-time MI, compared to older patients [11]. Emotional distress in this group was also linked to lower adherence to key secondary prevention behaviors (i.e., physical activity and smoking cessation). These findings underscore the need for psychological interventions tailored to the specific needs and contexts of younger people who have had an MI. Importantly, traditional cardiac rehabilitation programs are typically tested on older patients and therefore likely developed with older patient populations in mind [12]. This may limit both their actual effectiveness and their perceived relevance for younger individuals.

### Psychological and recovery-focused interventions

Traditional psychological interventions in cardiac rehabilitation often focus on stress management techniques aimed at reducing or reinterpreting stressors [13]. These interventions have small to moderate effects on symptoms of anxiety, depression, and stress, and their impact on CVD events is inconsistent [12]. An alternative approach emphasizes recovery processes, promoting strategies that enable patients to recover efficiently in post-stress situations. The stress-recovery program Balance in Everyday Life (BEL), which is grounded in cognitive‒behavioral principles, has shown significant benefits in improving recovery processes [14] and reducing stress, anxiety, and depression [15] in individuals with high levels of perceived stress. Such recovery-focused interventions could be particularly beneficial for adults with and history of MI at a younger age, as they build resilience rather than avoid stressors, helping individuals recover in a way that prevents stress from compromising their health. However, BEL has not yet been tested in this population.

### Internet-delivered psychological interventions in cardiac rehabilitation

To improve accessibility, internet-based psychological interventions have been proposed. These programs, which combine online content with therapist support, can address barriers such as geographical distance, stigma, and limited availability of therapists [16]. Internet-based CBT (iCBT) has shown promise for CVD patients [17]. A recent review and meta-analysis demonstrate that eHealth stress management interventions substantially improve psychologyical health parameters in patients with CVD [18]. However, the pooled standardized mean difference did not reach the commonly used threshold (0.5) for a medium effect size. Moreover, dropout rates in internet-delivered interventions tend to be high [19, 20], possibly partly because many patients prefer face-to-face therapy. Synchronous, video-based interventions may offer an alternative, although they are less studied [21].

### Significance of public involvement in this project

In this project, the PRPs provide insights essential for designing patient-centered interventions for people who experienced an MI at a younger age (< 55 years), who have unique psychological needs and rehabilitation challenges. By integrating PRP feedback at each stage of the research, we aim to create an intervention that reflects the real-life experiences and priorities of younger MI patients experiencing CD. Their involvement aims to ensure that our study materials, recruitment strategies, and intervention content are understandable, relevant, and accessible. Through this collaborative approach, we aim not only to enhance the scientific rigor of our research but also to increase the practical applicability of the findings, with the goal of delivering an intervention that is effective and perceived as relevant and supportive by those recovering from MI.

In this project, the PRPs mainly act as advisors, contributing their perspectives to a wide range of issues, including study design, outcome measures, recruitment strategies, and dissemination. While their role is advisory in nature, the breadth of their involvement means that some contributions also move toward what is described in the literature as co-design. This is particularly the case when influencing the adaptation of intervention content and study procedures, and, in one instance, contributing directly to coding and interpretation of data in parallel with a researcher. A recent bibliometric review reveals that while research on co-production in healthcare has expanded rapidly, the field remains relatively immature and fragmented, with limited empirical evidence on its actual impacts [22]. Co-creation in community-based health services has shown potential to enhance societal impact, provided that certain key principles are followed, such as placing human experience at the center, and maintaining strong collaborative processes and relationships [23]. Conceptual work has further emphasized that healthcare services are inherently co-produced, and that recognizing this principle is essential for effective collaboration with patients [24]. In cardiac care, empirical evidence shows that co-design processes can be used to develop more accessible and contextually adapted rehabilitation programs, particularly in underserved regions, thereby improving patient-centeredness and equity of service delivery (Bernier et al., 2024). Taken together, existing research highlights both the promise of more extensive forms of patient involvement within the broader framework of PPIE and the significant knowledge gaps that remain, particularly regarding psychological and behavioral interventions for people who experienced an MI before the age of 55.

### Best practices for patient and public involvement and engagement

To ensure high-quality public involvement in this project, we adhered to well-established guidelines and standards, including the GRIPP2 checklist for reporting patient and public involvement in health and social care research [25]. The GRIPP2 checklist emphasizes the importance of engaging public contributors, such as PRPs, from the early stages of research design through dissemination. Following these principles, we involve PRPs not only in the initial design phase but also throughout the entire lifecycle of the study, enabling a continuous, collaborative approach that is integrated at each stage.

Our approach also aims to go beyond standard PPIE practices by embracing elements of co-production, in which PRPs actively participate in data analysis and interpretation. This level of involvement, where PRPs contribute insights during data analysis and interpretation, is less commonly seen in prior PPIE-based research [26], but we believe that it is essential for creating patient-centered outcomes that truly reflect the lived experiences of MI survivors experiencing CD.

The project is further guided by the UK Standards for Public Involvement in Research, a practical tool developed to set hallmarks for effective PPIE [27]. These standards highlight several key principles that inform our approach: *Inclusive Opportunities* (e.g., we will recruit PRPs with diverse experiences and demographics), *working together* (e.g., regular meetings with a fixed group), *support and learning* (e.g., promote support and nonobligated learning opportunities continuously), c*ommunications* (e.g., using plain language), *impact* (e.g., continuously evaluate and document the impact of public involvement. In addition, we plan to include PRPs in terms of research management, leadership, and decision-making, ensuring that their voices are integral to guiding the project. By adhering to these standards, we aim to create a research process that is inclusive, respectful, and reflective of the perspectives of those it intends to serve. The active participation of PRPs, aligned with these best practices, is intended to foster interventions that are both effective and tailored to the real-life needs, preferences, and circumstances of people who experienced an MI at a younger age.

The theoretical underpinnings of PRP involvement draw from traditions in both co-design and participatory democracy, but are distinct in their emphasis on social justice, empowerment, and the systemic redistribution of power in research. Rather than solely providing input on practical aspects, PRPs are in several instances positioned as co-creators (for example coding data in parallel with a researcher) whose experiential knowledge complements scientific evidence and challenges prevailing assumptions about whose voice matters in knowledge production.

## Overall project purpose and aims

The overarching goal of this four-year project is to adapt the behavioral intervention *BEL* [15] for internet delivery to people who have experienced an MI under the age of 55 and experience CD. The project, conducted in close collaboration with PRPs, consists of three intervention studies and one public contribution study. The specific aims of the four studies are as follows:

- **Study 1**: Explore PRPs’ perceived impact on the design, implementation, and evaluation of the internet-delivered BEL (iBEL) intervention (in Study 2–4), and describe the process of their involvement in research activities.
- **Study 2**: Adapting the BEL program for internet delivery to MI survivors under 55 years of age with CD.
- **Study 3**: Test the feasibility of the iBEL intervention program adapted in Study 2.
- **Study 4**: Evaluate the clinical efficacy and cost-effectiveness of the adapted iBEL program.

Together, Studies 2–4 address the development, feasibility testing, and effectiveness evaluation of the intervention. Study 1 runs in parallel with all these studies and examines how patient research partners contribute to and influence the research process across all phases. Study 2 gathers input from people with lived experiences of a previous myocardial infarction and CD through focus group interviews to guide how the intervention is adapted for internet delivery. Study 3 tests the feasibility and acceptability of the adapted intervention using predefined progression criteria, including recruitment and retention rates, adherence levels, and participant feedback. Study 4 evaluates clinical effectiveness and cost-effectiveness using standardized patient-reported outcomes, such as measures of CD, stress recovery, and quality of life.

This paper presents the study protocol for Study 1, which explores PRPs’ perceived impact on research activities, including the design, implementation, and evaluation of the iBEL intervention, and describes the process of their involvement. By focusing on public contribution as a cornerstone of this project, Study 1 aims to assess how PRP engagement influences the overall research process and outcomes. This will provide valuable insights for future studies, particularly regarding optimal ways of involving patient research partners in psychological and behavioral intervention research for cardiovascular disease (CVD) patients.

## Methods

### Design

**Fig. 1:**
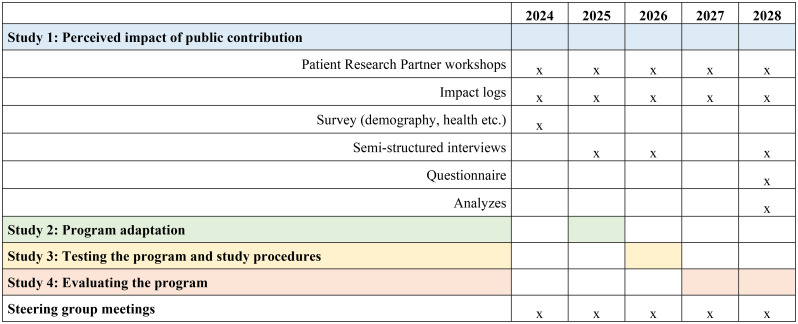
Timeline of Study 1 activities (2024–2028), including workshops, data collection, and analyses to evaluate the impact of Patient Research Partners. Colors indicate the timing of Studies 2–4. Steering group meetings involve all studies and take place annually; “x” marks occurrence each year

This prospective mixed methods PPIE study is designed to favor a collaborative process of knowledge production and co-learning, including people with lived experience of a previous myocardial infarction and CD in the research process. We follow the GRIPP2–Long Form (GRIPP2-LF) checklist [25] to guide and report on patient and public involvement activities in this study, with the completed checklist provided as Supplementary Table 1. In Fig. 1, the overall flow of the four interrelated studies, including the stages, workshops, and data collection points, and how this study (Study 1) runs in parallel with Studies 2–4, are illustrated.

**Table 1:** Preliminary topics for the workshops

All workshops
Main topics: Review accuracy of impact log; Reflect on project progress; Additional agenda items as needed; Summarize key insights and next steps • Evaluate the perceived accuracy of notes made in the impact log during the previous workshop. • The progression of the project results from potential tasks and reflections from PRPs since the last workshop. • Additional agenda items may be incorporated on the basis of the current status and challenges of the project. • The final segment of every workshop involves concluding the current workshop by summarizing key insights and eventual commitments for future actions.
Workshop 2:1* Main topic: Orientation to project and public contribution. • Inform the collaborators with the overarching research project and the public contribution part of the project. • Expectations are addressed and discussed, roles and responsibilities are defined, and PRP preferences concerning meeting times, workshop structure, and workshop content are explored. • Reflect on the overall project. • Considerations regarding the use of focus groups in Study 2 include reflecting on the facilitation of a psychologically safe and constructive atmosphere and considering a wide range of viewpoints as possible. • Reflect on the Cardiac Distress Inventory (CDI).
Workshop 2:2 Main topic: Feedback on preliminary focus group results. • Gather feedback on the preliminary results and experiences following the completion of two to three out of the four preliminary focus group meetings in Study 2. The emphasis is on reflecting on the interpretation of the preliminary analyses and, on the basis of these strategies, how to optimize the efficiency of the remaining upcoming focus group meetings. Furthermore, views regarding saturation will be discussed, as will whether more than the planned four focus group meetings are needed to achieve saturation. Workshop 2:3 Main topics: Current challenges (specific agenda will be decided later). Workshop 2:4 Main topics: How to adapt BEL for online delivery. • The preliminary findings, particularly the results, are reflected, and conclusions, including the implications of adapting BEL for people with lived experiences through internet delivery, are drawn.
Workshop 3:1 Main topics: Recruitment and retention strategies. • Reflect and advice regarding recruitment, retention, participant information sheets, and invitations, which involves exploring recruitment and retention procedures and methods, as well as examining written materials that participants will come across, such as the invitation letter, participant information sheet, and consent information. Workshop 3:2 Main topic: Assessment strategy. • This workshop will focus on assessment, which includes discussions on the quantity of measurement instruments, the timing and frequency of measurements, reminders for measurement adherence, alternative measurement tools, the exploration of measures beyond self-assessment methods, the determination of suitable methods for assessing work ability, the evaluation of face validity and the user-friendliness of measurement instruments, the assessment of their functionality for the current target group, and the addressing of ethical concerns.
Workshop 3:3 Main topics: Posttreatment interview guide; Recruitment results. • We gathered feedback on a preliminary posttreatment semi-structured interview guide. • The results of participant recruitment were reviewed, challenges that may have arisen were addressed, and advice regarding solutions if needed was provided. Workshop 3:4 Main topics: Feasibility and acceptability and planning for the evaluation study We reflect on preliminary findings, draw conclusions, and examine the acceptability and feasibility of the intervention. • Discussions on conducting the evaluation study for the target population, including addressing potential ethical concerns, are needed. Workshop 4:1 • See workshop 3:1. Workshop 4:2 Main topics: Challenges in recruitment and intervention delivery • Reflections and advice related to the challenges encountered in the research project, specifically those associated with the recruitment of participants and intervention delivery. Workshop 4:3 Main topics: Review pilot study results. • The primary aim is to examine the internal pilot study, with a specific focus on evaluating whether the criteria for progressing with the study have been met. If these criteria are fulfilled, the examination will be extended to determine whether any adjustments are warranted for the subsequent phase of the research.
Workshop 4:4 Main topics: Current challenges (specific agenda will be decided later). Workshop 4:5 Main topics: Preliminary results and implications of Study 1 and 4. • Preliminary results, conclusions, and implications regarding the clinical efficacy and cost-effectiveness of the intervention and the full project are discussed. • Dissemination of the project’s results and determination of the appropriate next steps from a research standpoint. • Discuss and advise potential ethical issues that may have arisen or may arise for the following stage of the research. • Preliminary results are discussed, conclusions drawn, and implications for study 1 (i.e., the public contribution aspect of the full project) are explored.

Note. * The initial number signifies the specific study within the project that will be addressed in the workshop, whereas the second number signifies its sequential order among the four workshops associated with each study

### Patient research partners

Five to eight PRPs will represent the target population across Sweden to form an advisory board. The PRPs provide feedback during most research activities. The target population is defined as individuals who have experienced an MI before the age of 55 years and subsequently experienced CD, with the event having occurred within the last five years. The advisory board will include both sexes and represent diverse demographics.

While suitability for the PRP or PCC roles is primarily assessed based on experience of MI and CD, demographic diversity, and ability to contribute, we also acknowledge the value of variation in personal characteristics such as communication style (e.g., more extroverted vs. more reserved individuals). Such variation can help ensure that a broad range of perspectives and needs are represented. Similarly, differences in eHealth literacy are expected within the target group, and we will pay attention to this aspect during the workshops and when interpreting PRP contributions.

PRPs will be invited to actively engage in thirteen online workshops. One of the PRPs serves as a public contribution coordinator (PCC) and is responsible for coordinating the work on the PRP’s advisory board. The PCC is also part of the steering group and represent the PRPs in research activities that do not allow the full group to participate (see details below). Suitability for the PRP or PCC roles is assessed by researchers based on factors such as relevant experience, demographic diversity, variation in experiences related to the care they received for their MI and CD, and fluency in Swedish.

PRPs are recruited through advertisements in the patient organization Heart and Lung Association’s (HLA; Riksförbundet HjärtLung in Sweden) magazine *Status* and their social media platforms. Additionally, recruitment is done via advertisement posted on the research group website, placed in nationwide digital editions of newspapers and on social media platforms, including Facebook and Instagram. The advertisement text may also be used on posters and flyers distributed at heart clinics in hospitals within the Uppsala–Stockholm area. People interested in participating can access detailed information about what the role of PRP entails and about the project via a secure website (i.e., REDCap) hosted by Uppsala university. The website link and a QR code are provided in the advertisements, allowing individuals to register their interest. The candidates provide basic demographic information. Following this, they are invited to a semi-structured recruitment interview outlining the research partnership’s purpose, activities, and responsibilities. The interviews cover topics such as the participant’s location, employment status (if applicable), MI experience, including CD and receiving health care, and fluency in both spoken and written Swedish. A comprehensive assessment is conducted by the researchers to determine the interviewees’ suitability for the role of the PRP or PCC. Those assessed as suitable and who remain interested are asked to digitally sign informed consent.

Throughout the project, the research team and PCC aim to create a supportive and open atmosphere in which PRPs feel comfortable sharing any concerns if participation in the project affects them negatively. Participants are not actively screened for wellbeing and are treated as fully capable adults; the intention is to respect their autonomy and integrity rather than to signal that they are overly vulnerable. Overly frequent or invasive questioning is avoided, as this could unintentionally suggest that negative effects are expected or create perceptions of risk. PRPs are encouraged to communicate any issues voluntarily and can decline tasks or withdraw from workshops at any time without consequences, maintaining full control over their participation.

One previous PRP (Pelle Johansson) initiated the overall project (of which this protocol describes one part) and played a key role in shaping the overall research idea and aims. The focus on people who experienced an MI at a younger age emerged from his long-term experiences as a PRP in many previous projects through his engagement in the HLA. However, before the first draft of this manuscript, he withdrew from the project due to retirement. As described above, one of the first actions in the present project was to start recruiting PRPs and, from them, a PCC (co-author HN). For the present article, only the PCC is co-author. In the future, as the PRP panel consolidates, all PRPs will be invited to co-author publications related to the project, based on the nature and extent of their contributions.

The involvement structure described here, including the workshop format and the role of the PCC, was initially developed by the academic team with input from PRP Pelle Johansson during the funding application stage. However, these plans were explicitly formulated as provisional and intended to be reviewed and adapted in collaboration with PRPs. This approach reflects a commitment to co-production, in which patient involvement is not only embedded throughout the research process but also allowed to shape the involvement model itself. As recommended in the GRIPP2-LF guidance, this reflexive and iterative stance is intended to support more authentic, context-sensitive, and responsive patient engagement.

PRPs are also offered the opportunity to meet peers in similar situations, which may provide additional support and perspective, particularly for younger MI patients who often only meet older patients in routine care. The research team remains flexible and responsive to PRPs’ preferences, adapting the format or focus of workshops within the scope of the project’s objectives.

### Public contributor coordinator

One PCC (HN), external to our research team, was recruited through the same procedures as the PRPs (see above). In addition to the questions asked to PRP candidates, PCC candidates were also asked about work experience relevant to the PCC role, such as leading meetings. The PCC coordinates and acts as the primary leader of the workshops. The PCC plays a pivotal role during the workshops, actively involving all the PRP members in discussions, guiding the group’s attention to current issues and topics for today’s workshop, and structuring breaks efficiently. The PCC is committed to fostering informal social interactions, aiming to contribute to a pleasant and engaging atmosphere, which previous research has highlighted as important for encouraging open communication [28]. Responsibilities include maintaining communication with the PRPs (e.g., via telephone, email, online communication platforms, or SMS) as well as handling the distribution of any required materials. Additionally, the PCC informs the steering group how the work and activities are progressing and being receptive to the research team’s views. The PCC participates in the research team´s steering meetings, scheduled approximately four times per year, with each session lasting approximately 90 min at a time convenient for the PCC, who may have daytime work. The inclusion of the PCC aims to ensure the representation of perspectives from the PRPs.

The PCC role is voluntary and reimbursed for time and travel. It is a pivotal position: the PCC coordinates the PRP advisory board, leads workshops, facilitates discussion, and represents PRPs in steering group meetings. The research team supports the PCC and maintains communication while respecting their autonomy, ensuring the role sustains patient engagement rather than serving as administrative assistance.

#### Workshops

The PRPs are scheduled to meet for four workshops of approximately three hours per Studies 2 and 3 and five workshops for Study 4. These structured workshops focus on the research content itself, such as study materials, protocols, and intervention design, rather than on governance or administrative matters, which are addressed in steering group meetings. The workshops take place online via the latest version of the Zoom platform (Zoom Video Communication) using end-to-end encryption. The preliminary topics for each workshop are listed in Table 1. These topics remain preliminary, as the perspectives of the PRPs may lead to revisions. All suggestions from the PRPs will be considered and weighed against practical limitations, high-quality research methodology and evidence-based treatments. The following strategies are used to reduce the risk of memory bias: multiple data sources (e.g., impact logs, semi-structured interviews, and questionnaires) and providing contextual stimuli by talking about experiences during workshops to aid accurate recall.

### Withdrawal

This is a multi-year study involving continuous PRP engagement, and participant turnover is anticipated. If a PRP misses several workshops or meetings, the PCC or a researcher will contact the person by telephone or in an online meeting to discuss the reasons for nonattendance and assess their motivation and ability to continue in the role. If the person chooses to leave the project or continued engagement is not feasible, a replacement may be recruited to maintain group function and diversity.

The recruitment process therefore remains open throughout the study to ensure continuity, representation, and adaptability. In addition to replacing withdrawn PRPs, we may also invite new participants if it becomes evident that further perspectives would enhance the relevance or scope of the study. The initial number of five to eight PRPs is a pragmatic starting point rather than a fixed limit.

If fewer than five PRPs remain in the project, new recruitment will be initiated. Similarly, if the PCC withdraws, a replacement will be recruited either from outside the project or from the existing PRP group. To minimize disruptions, workshops are rescheduled if more than two PRP or the PCC is unable to attend. Individual follow-up meetings may also be arranged depending on project needs and available resources.

### Data collection

#### Survey

Immediately after providing informed consent, the PRPs and the PCC are required to complete a survey, providing information on demographic data and health data, such as age, sex, education level, employment status, years since MI, severity of CD, stress, general anxiety, depression, recovery processes, quality of life, presence of comorbid physical and mental health conditions, and geographic location. This information guides the interpretation of PRP feedback and support diversity monitoring.

#### Impact log

An impact log is recorded during and after each workshop [29]. The impact log includes (1) the date and time of the workshop; (2) those who were present and who were absent and reasons for absence; (3) the planned agenda of the workshop; (4) notes of revisions of impact log posts from previous workshops; and (5) ideas, opinions and (6) suggestions from PRPs (there are no requirements for consensus within the group). Important differences in the opinions and perceptions of those who have the most influence regarding ideas, suggestions, corrections for accuracy, implementation, and other observations of importance will be noted.

Later, this is compared to what has happened in the study, and a judgment will be made as to whether this followed the PRP’s suggestions. The reasons for implementing or not implementing ideas or suggestions from the PRPs are also be noted. Workshop discussions are audio-recorded for the possibility of checking the accuracy of notes in the impact log.

A designated researcher is responsible for drafting the impact log after each workshop. The draft is then reviewed within the PCC to ensure completeness. At the beginning of each subsequent workshop, PRPs are invited to review the log entries to confirm accuracy and provide corrections or additions as needed.

In addition to recording suggestions and feedback, the impact logs will also capture instances where PRP contributions were challenging to implement, less effective, or highlighted difficulties in the engagement process. The log will also capture whether suggestions were supported by one or several PRPs, providing a rough indication of the level of agreement within the group.

#### Semi-structured interviews

We will conduct semi-structured interviews with all the PRPs and the PCC face-to-face or via an online meeting immediately after studies 2–4, respectively. The interviews will be audio-recorded. Interviews will explore not only the perceived contributions of PRPs but also any challenges or aspects of the involvement process that were less effective, to provide a more balanced understanding of patient engagement.

#### Questionnaire

Toward the conclusion of the project, but before workshop 4:5, a questionnaire adapted from the framework developed by Mann et al. [29] will be administered to the PRPs and PCCs. Example questions include: “Please describe one way in which PRP input has led to a concrete change in the study (e.g., intervention content, recruitment materials, or procedures)”, “In your view, what has been the most important contribution of the PRP group to the project?” and “What aspects of the involvement process have facilitated or hindered your ability to contribute meaningfully?”. This questionnaire will specifically capture both positive contributions and challenges experienced by PRPs and PCCs, supporting a balanced reflection on the involvement process. The surveys will also allow for descriptive summaries of PRPs’ views on key aspects of the involvement process, providing complementary quantitative indicators.

#### Data analysis

The analysis of data is primarily performed by the researcher involved in the workshops. Additionally, all members of the research team and the PCC work systematically through the full data sets, ensuring that each data item receives equal attention. Additionally, the PRPs will be encouraged to reflect on and discuss the initial findings during workshops 4:5. The researcher remains receptive to feedback and be willing to adjust and refine the analyses based on these reflections and discussions.

Data from all sources, i.e., (a) impact logs, (b) semi-structured interviews, and (c) questionnaires, will be thematically analyzed following the six steps of thematic analysis suggested by Nowell et al. [30]: (1) familiarizing yourself with the data, (2) generating initial codes, (3) searching for themes, (4) reviewing themes, (5) defining and naming themes, and (6) producing the report. These steps are derived from the trustworthiness criteria outlined by Lincoln and Guba [31], which concern (1) credibility, (2) transferability, (3) dependability and (4) confirmability. Additionally, an audit trail is maintained, documenting data collection instruments, raw data, the data analysis process, procedures, and decision-making steps for transparency and accountability. Finally, we document the extent to which PRP suggestions are implemented and jointly reflect with the PRPs on how their input aligns with key study decisions. This process will support reflexivity and transparency regarding the role and impact of patient involvement. The thematic analysis will explicitly include identification of both effective aspects and challenges in the PRP approach, ensuring that the final report provides a balanced view of patient involvement.

In addition to the qualitative analyses, we will also explore simple quantitative indicators. This includes tracking levels of agreement/disagreement in survey responses, as well as recording the proportion of PRP suggestions noted in the impact log that were subsequently implemented. These indicators will not be treated as definitive measures of impact but will complement the qualitative findings and provide additional transparency.

#### Reporting

The results will be reported primarily in international peer-reviewed journals and at scientific conferences. We will report PRP activities and perceived impact in accordance with the GRIPP2 checklist [25] and qualitative results in accordance with the Standards for Reporting Qualitative Research checklist [32]. 

#### Ethics

We supply PRPs with comprehensive written information detailing the project, particularly the responsibilities associated with participation. The information includes contact for the Principal Investigator (PI). Potential participants will be explicitly informed that their participation is voluntary and that they can withdraw from the study at any point without having to tell why and without facing any negative consequences. Participants are reimbursed for their time (200 SEK [~ 20 EUR] per hour), and transportation and parking costs are also reimbursed when used. Written informed consent is required from all individuals to participate as a PRP or PCC.

Given the longitudinal nature of the study, it is ethically important to allow PRPs to discontinue their participation without consequence should their circumstances change. To uphold both the ethical principle of voluntariness and the scientific quality of the study, procedures are in place to recruit additional PRPs if needed due to participant withdrawal or to enhance representation. This ensures that participation remains sustainable for individuals and that the advisory board continues to function effectively and reflect a diversity of experiences throughout the study period.

We also recognize that the PCC role carries particular responsibility and therefore a potential risk of overburdening the individual. To mitigate this risk, the PCC is continuously supported by the research team (administrative assistance, flexible scheduling, and regular check-ins), and responsibilities can be redistributed to researchers or other PRPs if necessary. Importantly, both the PCC and the PRPs are explicitly given the opportunity to decline tasks they do not wish to take on, and this decision control is emphasized throughout the project. Should the workload nonetheless become unmanageable, we have procedures in place for recruiting an additional or replacement PCC.

All the data collected, e.g., via questionnaires, impact logs, audio recordings of workshop discussions, and transcriptions of semi-structured interviews, are processed in strict accordance with the General Data Protection Regulation (EU 2016/679). To ensure security, this information are stored in locked fireproof cabinets and/or on secure servers hosted by Uppsala university. The research follows the Declaration of Helsinki. We have received ethical approval to conduct the study from the Regional Ethics Committee in Uppsala, Sweden (Dnr 2024-01887-01).

## Discussion

This project aims to adapt and test an existing stress recovery program, the BEL, for online delivery to individuals under 55 years of age who have survived an MI and experienced CD. This protocol outlines one of four substudies, with the overarching goal of engaging PRPs in the design, adaptation, and evaluation phases of the intervention (i.e., Studies 2–4). The primary aim of Study 1 is to explore PRPs’ perceived impact on the design, implementation, and evaluation of the iBEL intervention, and to describe the process of their involvement in research activities. This focus on public contribution aligns with broader trends in health research, where PPIE is increasingly recognized as key to enhancing the relevance and quality of research outcomes. By systematically involving PRPs in all substudies, we aim to ensure that the intervention remains patient-centered, which is vital for both efficacy and practical implementation.

We have followed established guidelines for engaging PRPs in our research project. To our knowledge, this is the first psychological intervention project for people with MI experiences which actively involves the target population across multiple phases, from intervention development to evaluating efficacy. PRPs’ involvement allows for a more patient-centered approach, ensuring that the intervention is not only scientifically sound but also practical and relevant to those with a history of MI and CD. A further unique feature of this study is the integration of people from the target group at two levels: first, through focus groups that provide feedback on the intervention’s adaptation, and second, by engaging PRPs in offering suggestions for conducting and interpreting the data from the focus group study itself.

### Public contributions and research relevance

Public contributions can significantly enhance the relevance and acceptability of health interventions, especially when people with lived experiences of a previous myocardial infarction and CD are involved in the early stages of development. PRPs can provide nuanced insights that researchers may overlook, contributing to better-designed interventions that more effectively address patients’ needs. Systematic data collection, including impact logs, interviews, and questionnaires, also allowed identification of challenges and less effective aspects of PRP involvement, ensuring a more balanced understanding of patient contributions. In this study, the involvement of PRPs not only ensures the intervention’s relevance but also helps identify potential barriers to implementation and retention, such as accessibility and the usability of online platforms. This is particularly important for people who have experienced an MI at a relatively young age, as they often face higher psychosocial challenges, such as work demands, compared with older individuals.

This model can be situated within the broader field of participatory research, where public contributors are increasingly recognized as active partners rather than passive informants. In line with previous frameworks of PPIE, our approach underscores how PRPs can meaningfully shape intervention design while also highlighting the need for structured processes (e.g., impact logs, interviews, and questionnaires) to capture their contributions. By grounding the work in participatory research theory, the study illustrates how patient input can be systematically integrated in ways that strengthen both the practical and scientific value of the research.

Notably, while public contribution enhances the study’s relevance, it must be carefully balanced with evidence-based practice. There is a potential risk of overemphasizing patient perspectives at the expense of scientific rigor. To mitigate this, we aim to allow PRPs to influence aspects of the study that align with existing evidence while carefully considering feedback that may diverge from established findings.

### Limitations and challenges

Several limitations should be acknowledged. First, the representativeness of the PRP group may be limited. Those who choose to participate might differ in important ways from the broader patient population, potentially skewing the findings. To address this, we aim to recruit a heterogeneous group of PRPs with diverse demographics and experiences related to MI and CD. Additionally, while a random sample is not feasible, we will explicitly acknowledge this limitation when interpreting the results.

Second, memory bias may affect PRPs’ recall of past experiences and influence their recommendations during the study. To mitigate this, we implement several measures (e.g., avoiding wording that suggests expected responses, and encourage PRPs to reflect on their experiences to help stimulate more accurate recall). Even with these measures, we must be cautious in interpreting findings influenced by retrospective data.

A third challenge, and a necessary complexity, is the heterogeneity of the PRP group, as individuals with diverse backgrounds may have differing perspectives. This variability may complicate synthesis, but it also enriches the findings and supports intervention flexibility. Managing such diversity is a critical aspect of developing psychological interventions that must be adaptable to different patient experiences and contexts. In this study we recognize heterogeneity and use it as an opportunity to refine the intervention, ensuring that it meets a broader range of needs.

Fourth, communication between researchers and PRPs presents a potential challenge. To address this, we use clear, jargon-free language supplemented by visual aids and provide training to both the PRP and the external PCC. Continuous feedback loops will be established to ensure ongoing dialogue and understanding throughout the research process.

Fifth, sustained PRP involvement include challenges related to shifting roles and power dynamics over time, uneven participation due to life circumstances, and the tension between representativeness and depth of involvement. The study seeks to balance these challenges by providing flexible participation structures, supporting continuity through the PCC role, and being transparent about the limits of representativeness.

Sixth, the overall structure for patient involvement was developed by the academic team prior to the recruitment of the broader PRP group. Although the project was initiated based on input from a highly experienced PRP and the involvement framework was intentionally designed to be provisional, this still represents a deviation from best practice as outlined in GRIPP2, where early co-design of PPIE procedures is recommended. Future studies may benefit from involving multiple PRPs already in the planning of involvement structures, particularly to enhance transparency, shared ownership, and contextual sensitivity from the outset.

Seventh, the PCC role itself may be a source of limitation. While the PCC provides important coordination and leadership in sustaining PRP involvement, this role also carries a risk of overburdening the individual. Given our ambition to integrate elements of co-design, there is an inherent tension between encouraging extensive contributions and ensuring that the workload remains ethically acceptable. We mitigate this by offering continuous support, allowing tasks to be redistributed when needed, and providing ongoing opportunities for the PCC and PRPs to decline activities they do not wish to engage in. Nevertheless, the potential risk of uneven workload remains a challenge, and future studies may need to explore additional strategies for balancing meaningful co-design with participant well-being.

The inclusion of structured methods to capture challenges through impact logs, interviews, and questionnaires provides a systematic basis for reflecting on less effective aspects of PRP involvement, complementing observations of successes and supporting a more balanced evaluation of patient engagement.

## Conclusion

This project represents a novel attempt to integrate patient involvement systematically throughout the entire research project, from design to evaluation of a novel psychological intervention for young MI patients. By actively engaging PRPs in all phases, this study aims to explore both the relevance and quality of the intervention for people who have experienced an MI at a younger age and who are experiencing CD. Although challenges related to memory bias, representativeness, and communication are anticipated, these challenges are being addressed through multiple strategies aimed at maintaining the rigor of the study. The findings will contribute not only to the development of a novel stress recovery intervention, the iBEL, but also to broader knowledge about the role of the public in intervention research. This study is pioneering in its combination of the use of PRPs in the study of the development and testing of a psychological intervention tailored specifically for young cardiac patients. Our aim is to make a significant contribution by bridging the gap between the psychological needs of this population and the knowledge of effective ways to address them. While the structure for patient involvement was intentionally designed to be flexible and open to revision, a limitation is that the broader group of PRPs was not engaged in shaping it. Future studies within the project may benefit from including PRPs already in the planning.

## Supplementary Information

Below is the link to the electronic supplementary material.

Supplementary Material 1

## Data Availability

Due to ethical and confidentiality considerations, the full qualitative data set cannot be shared. However, summarized findings and selected anonymized excerpts may be made available upon reasonable request.
