# Supplementary material for: Involving people with lived experiences in the study of a behavioral stress-recovery e-intervention for myocardial infarction patients younger than 55 with cardiac distress: a study protocol
Source: Res Involv Engagem. 2025 Oct 27;11:126. doi: 10.1186/s40900-025-00795-z (PMC12560494; doi:10.1186/s40900-025-00795-z)
Supplement: Supplementary file 1 — Supplementary Material 1 [file 40900_2025_795_MOESM1_ESM.pdf]

Supplementary Table 1

GRIPP2 long form

Section and topic	Item	Reported on page No (Start-line No)
**Section 1: Abstract of paper**		
1a: Aim	Report the aim of the study	7 (130)
1b: Methods	Describe the methods used by which patients and the public were involved	7 (134)
1c: Results	Report the impacts and outcomes of PPI in the study	N/A
1d:Conclusions	Summarise the main conclusions of the study	7 (143)
1e: Keywords	Include PPI, "patient and public involvement," or alternative terms as keywords	8 (147)
**Section 2: Background to paper**		
2a: Definition	Report the definition of PPI used in the study and how it links to comparable studies	9 (162)
2b: Theoretical underpinnings	Report the theoretical rationale and any theoretical influences relating to PPI in the study	9 (167)
2c: Concepts and theory development	Report any conceptual models or influences used in the study	12 (241)
**Section 3: Aims of paper**		
3: Aim	Report the aim of the study	14 (296)
**Section 4: Methods of paper**		
4a: Design	Provide a clear description of methods by which patients and the public were involved	17 (350)
4b: People involved	Provide a description of patients, carers, and the public involved with the PPI activity in the study	16 (137)
4c: Stages of involvement	Report on how PPI is used at different stages of the study	20 (427)
4d: Level or nature of involvement	Report the level or nature of PPI used at various stages of the study	17-20 (350)
**Section 5: Capture or measurement of PPI impact**		
5a: Qualitative evidence of impact	If applicable, report the methods used to qualitatively explore the impact of PPI in the study	23 (504)
5b: Quantitative evidence of impact	If applicable, report the methods used to quantitatively measure or assess the impact of PPI	24 (525)
5c: Robustness of measure	If applicable, report the rigour of the method used to capture or measure the impact of PPI	23 (504)
**Section 6: Economic assessment**		
6: Economic assessment	If applicable, report the method used for an economic assessment of PPI	N/A
**Section 7: Study results**		
7a: Outcomes of PPI	Report the results of PPI in the study, including both positive and negative outcomes	N/A
7b: Impacts of PPI	Report the positive and negative impacts that PPI has had on the research, the individuals involved (including patients and researchers), and wider impacts	N/A
7c: Context of PPI	Report the influence of any contextual factors that enabled or hindered the process or impact of PPI	N/A
7d: Process of PPI	Report the influence of any process factors, that enabled or hindered the impact of PPI	N/A
7ei: Theory development	Report any conceptual or theoretical development in PPI that have emerged	N/A
7eii: Theory development	Report evaluation of theoretical models, if any	N/A
7f: Measurement	If applicable, report all aspects of instrument development and testing (eg, validity, reliability, feasibility, acceptability, responsiveness, interpretability, appropriateness, precision)	N/A
7g: Economic assessment	Report any information on the costs or benefit of PPI	N/A
**Section 8: Discussion and conclusions**		
8a: Outcomes	Comment on how PPI influenced the study overall. Describe positive and negative effects	N/A
8b: Impacts	Comment on the different impacts of PPI identified in this study and how they contribute to new knowledge	N/A
8c: Definition	Comment on the definition of PPI used (reported in the Background section) and whether or not you would suggest any changes	NA
8d: Theoretical underpinnings	Comment on any way your study adds to the theoretical development of PPI	26 (588)
8e: Context	Comment on how context factors influenced PPI in the study	26 (588)
8f: Process	Comment on how process factors influenced PPI in the study	26 (588)
8g: Measurement and capture of PPI impact	If applicable, comment on how well PPI impact was evaluated or measured in the study	NA
8h: Economic assessment	If applicable, discuss any aspects of the economic cost or benefit of PPI, particularly any suggestions for future economic modelling.	NA
8i: Reflections/critical perspective	Comment critically on the study, reflecting on the things that went well and those that did not, so that others can learn from this study	27 (614)
